# Serotyping, antibiotic susceptibility, and virulence genes screening of *Escherichia coli* isolates obtained from diarrheic buffalo calves in Egyptian farms

**DOI:** 10.14202/vetworld.2017.769-773

**Published:** 2017-07-11

**Authors:** Ashraf S. Hakim, Shimaa T. Omara, Sohier M. Syame, Ehab A. Fouad

**Affiliations:** Department of Microbiology and Immunology, National Research Centre, Dokki, Cairo, Egypt

**Keywords:** antibiotic resistance determinants, buffalo, Egypt, *Escherichia coli*, virulence

## Abstract

**Aim:**

In Egypt as in many other countries, river water buffalo (*Bubalus bubalis*) is considered an important source of high-quality milk and meat supply. The objective of this study was to investigate serotypes, virulence genes, and antibiotic resistance determinants profiles of *Escherichia coli* isolated from buffalo at some places in Egypt; noticibly, this issue was not discussed in the country yet.

**Materials and Methods:**

A number of 58 rectal samples were collected from diarrheic buffalo calves in different regions in Egypt, and bacteriological investigated for *E. coli* existence. The *E. coli* isolates were biochemically, serologicaly identified, tested for antibiotic susceptibility, and polymerase chain reaction (PCR) analyzed for the presence of antibiotic resistance determinants and virulence genes.

**Results:**

Overall 14 isolates typed as *E. coli* (24.1%); 6 were belonged to serogroup O78 (10.3%), followed by O125 (4 isolates, 6.9%), then O158 (3 isolates, 5.2%) and one isolate O8 (1.7%), among them, there were 5 *E. coli* isolates showed a picture of hemolysis (35.7%). The isolates exhibited a high resistance to β lactams over 60%, followed by sulfa (50%) and aminoglucoside (42.8%) group, in the same time the isolates were sensitive to quinolone, trimethoprim-sulfamethoxazole, tetracycline (100%), and cephalosporine groups (71.4%). A multiplex PCR was applied to the 14 *E. coli* isolates revealed that all were carrying at least one gene, as 10 carried *bla*TEM (71.4%), 8 *Sul*1 (57.1%), and 6 *aad*B (42.8%), and 9 isolates could be considered multidrug resistant (MDR) by an incidence of 64.3%. A PCR survey was stratified for the most important *E. coli* virulence genes, and showed the presence of Shiga toxins in 9 isolates carried either one or the two *Stx* genes (64.3%), 5 isolates carried *hyl*A gene (35.7%), and *eae* in 2 isolates only (14.3%), all isolates carried at least one virulence gene except two (85.7%).

**Conclusion:**

The obtained data displayed that in Egypt, buffalo as well as other ruminants could be a potential source of MDR pathogenic *E. coli* variants which have a public health importance.

## Introduction

*Escherichia coli* is a member of the genus *Escherichia* within the family *Enterobacteriaceae*. Members of this family are vastly extended in the environment. *E. coli* is ordinarily a non-pathogenic member of the animal intestinal flora. However, certain strains have developed virulence and antibiotic resistance factors, so may cause a variety of infections in humans and animals, strains of *E. coli* that cause enteric disease are stated enterovirulent or diarrheagenic *E. coli* (DEC) [1].

Ruminants represent a major reservoir for pathogenic *E. coli* access into the human communities through the food chain [2]. Diagnosing DEC in humans, food, and the environment has revealed that non-O157:H7 serotypes are more responsible for extreme infections in humans, and considered to be of greater clinical significance as causes of human disease than O157 strains in different countries [3].

Presently, the rising of antimicrobial resistance is a public health concern, in particular the use and misuse of antimicrobials, especially in animal population as growth promoters. The main factor in the emergence of multidrug-resistance (MDR) strains is the ability of bacteria to gain and disseminate exogenous genes through mobile genetic elements [4]. The upgrowth of *E. coli* isolates with MDR phenotypes has been formerly reported and may extend from one ecosystem to another by lateral gene transfer; integrons [5].

The pathogenic *E. coli* usually possess abundant virulence determinants that share in the disease pathogenesis; Shiga toxin-producing *E. coli* (STEC) represents a substantial emerging set of food-borne pathogens, as farm animals, particularly bovine, have been incriminated as natural reservoirs of STEC worldwide [6]. Attaching and effacing (A/E) *E. coli* are characterized by their ability to cause A/E lesions in the gut mucosa of human and animal hosts leading to diarrhea [7].

Recently, the studies concerned the virulence genes and serotypes of *E. coli* isolated from different animal and environmental sources in Egypt take a some attention [8-10], “but not yet in buffalo which is considered better than cattle in accommodation with the Egyptian climate conditions and distributed either in solitary populations or small holder farms [11].”

In Egypt, like many developing countries, there are commonly used antibiotics in human as well as the farm animal population as aminoglycosides (gentamicin, tobramycin, kanamycin, …etc.), β-lactam (ampicillin, amoxicillin, etc.), and sulfa groups due to their low costs, so investigation of their resistance genes is of public concern. Although there were many researches over the world implicated ruminants, especially cattle, as a principal reservoir of enterovirulent *Escherichia coli*, only a few reports on the role of buffalo as a reservoir of various *E. coli* pathotypes.

## Materials and Methods

### Ethical approval

As per CPCSEA guidelines, a study involving clinical samples does not require the approval of the Institute Animal Ethics Committee.

### Bacterial isolation and identification

Rectal samples were collected from 58 diarrheic buffalo calves in sterile swabs, dipped into trypticase soya broth were and incubated aerobically overnight at 37°C.

Samples were streaked onto MacConkey sorbitol agar (oxoid); then, the lactose fermenting pink colonies were picked and cultured onto eosin methylene agar (oxoid) and incubated as described above. The green metallic colonies were picked and examined for their biochemical characters; API 20E kit (Bio Merieux) was performed according to manufacturer’s instruction to detect the biochemical profile of the isolated organisms.

### Antimicrobial susceptibility testing

Phenotypical antibiotic susceptibility was tested applying the disk diffusion method on Mueller–Hinton agar plates (oxoid) according to the guidelines of Clinical and Laboratory Standards Institute [12]. A panel of 15 antibiotic discs was used as follows: Cefotaxime (30 μg), cefazolin (30 μg), trimethoprim/sulfamethoxazole (25 μg), sulphaprim (50 μg), ampicillin (10 μg), amoxicillin (10 μg), erythromycin (15 μg), amikacin (30 μg), kanamycin (30 μg), gentamycin (10 μg), ciprofloxacin (5 μg), norfloxacin (10 μg), cefadroxil (30 μg), chloramphenicol (10 μg), and tetracycline (10 μg).

### Serological identification of E. coli isolates

The serological typing based on agglutination reactions of the somatic antigens (O). O antigens were identified as described by Guinée *et al*. [13]. All available somatic antigens were (O1 to O185) antisera which done in Animal Health Institute Laboratory, Dokki, Giza.

### Hemolysis assay

*E. coli* isolates were propagated on blood agar base (oxoid) supplemented with 5% washed sheep erythrocytes. The plates were incubated at 37°C for 24 h, and the presence of a clear colorless zone surrounding the colonies indicated b-hemolytic activity [14].

### DNA extraction

DNA extraction from the samples was performed using the QIAamp DNA Mini kit (Qiagen, Germany, GmbH) with modifications from the manufacturer’s recommendations. Briefly, 200 µl of the sample suspension was incubated with 10 µl of proteinase K and 200 µl of lysis buffer at 56°C for 10 min. After incubation, 200 µl of 100% ethanol was added to the lysate. The sample was then washed and centrifuged following the manufacturer’s recommendations. Nucleic acid was eluted with 100 µl of elution buffer provided in the kit.

### Oligonucleotide primer

Primers used were supplied from Metabion (Germany) are listed in Tables-1 [15-17] and 2 [18-20].

**Table-1:** Multiplex PCR: Primers sequences, target genes, amplicon sizes, and cycling conditions.

Target gene	Primers sequences	Amplified segment (bp)	Primary denaturation	Amplification (35 cycles)			Final extension	Reference

				Secondary denaturation	Annealing	Extension		
*aadB*	GAGCGAAATCTGCCGCTCTGG	319	94°C 5 min	94°C 30 s	55°C 45 s	72°C 45 s	72°C 10 min	[15]
	CTGTTACAACGGACTGGCCGC							
*blaTEM*	ATCAGCAATAAACCAGC	516						[16]
	CCCCGAAGAAC GTTTTC							
*Sul1*	CGGCGTGGGCTACCTGAA CG	433						[17]
	GCCGATCGCGTGAAGTTC CG							

PCR=Polymerase chain reaction

**Table-2:** Diplex PCR: Primers sequences, target genes, amplicon sizes, and cycling conditions.

Target gene	Primers sequences	Amplified segment (bp)	Primary denaturation	Amplification (35 cycles)			Final extension	Reference

				Secondary denaturation	Annealing	Extension		
*Stx1*	ACACTGGATGATCTCAGT GG	614	95°C 3 min	94°C 60 s	59°C 45 s	72°C 90 s	72°C 10 min	[18-20]
	CTGAATCCCCCTCCATTA TG							
*Stx2*	CCATGACAACGGACAGCAGTT	779						
	CCTGTCAACTGAGCAGCACTTTG							
*hylA*	ACGATGTGGTTTATTCTG GA	165			53°C 45 s			
	CTTCACGTGCCATACATAT							
*eae A*	GACCCGGCA ACAAGCATA	384						
	AGC							
	CCACCTGCAGCAACAAGAGG							

PCR=Polymerase chain reaction

### Polymerase chain reaction (PCR) amplification

Primers were utilized in a 50-µl, PCR reaction containing 25 µl of EmeraldAmp Max PCR Master Mix (Takara, Japan), 1 µl of each primer of 20 pmol concentrations, 9 µl of water, and 10 µl of DNA template. The reactions were performed either multiplex (*aad*B, *bla*TEM, and *Sul*1) or diplex (*Stx*1 and *Stx*2) or (*hyl*A and *eae* A) as described in the Tables-1 and 2 in an applied biosystem 2720 thermal cycler.

### Analysis of the PCR products

The products of PCR were separated by electrophoresis on 1.5% agarose gel (Applichem, Germany, GmbH) in ×1 Tris/Borate/ethylenediaminetetraacetic acid buffer at room temperature using gradients of 5 V/cm. For gel analysis, 30 µl of the products was loaded in each gel slot. A 100 bp ladder (Qiagen, Germany, GmbH) was used to determine the fragment sizes. The gel was photographed by a gel documentation system (Alpha Innotech, Biometra) and the data were analyzed through computer software.

Reference *E. coli* strain ATCC35150 (O157:H7, stx1, stx2, eae, hylA), and *E. coli* HB 101 inv+ were used as a positive control and *S. aureus* ATCC29737 as a negative control.

## Results and Discussion

### The isolation and serotyping of E. coli

Of 58 collected rectal samples, there were 14 typed isolates as *E. coli* (24.1%); 6 were belonged to serogroup O78 (10.3%), followed by O125 (4 isolates, 6.9%), then O158 (3 isolates, 5.2%) and one isolate O8 (1.7%), only 5 *E. coli* isolates showed a picture of hemolysis (35.7%). These results were some extent close to that obtained by Beraldo *et al*. [6] who determined the prevalence of enteropathogenic *E. coli* and STEC *E. coli* among buffalo and found that 76 out of 256 samples (29.7%) were positive, somewhat approach another study applied on fecal samples from 174 slaughter buffalo in Dhaka, Bangladesh, as 37.9% [21]. On the other side, there were other studies showed a higher incidences; Oliveira *et al*. [22] reported the presence of *E. coli* in water buffaloes in Brazil “as the first survey in South America” ranged from 0 to 64%, depending on the farm. However, in another study in Italy, the incidence of isolation reached 70% (220/314) among Mediterranean water buffalo calves <4 weeks old affected by severe diarrhea with a lethal outcome [23]; moreover, the same incidence was recorded in buffalo farms, in the central region of Vietnam [24].

The serotyping of the study isolates showed three serogroups O8, O125, and O158, which also detected among 36 different O groups in another study [21], the fourth serotype represented O78 which was commonly found in cattle population in Egypt [9,10].

The antibiotic sensitivity assay showed that the *E. coli* isolates were more frequently resistant to ampicillin (71.4%), amoxicillin (64.3%), followed by intermediate resistance to sulphaprim (50%) then gentamicin and kanamycin (42.8%), while the isolates were found to be highyly sensitive to ciprofloxacin, norfloxacin, trimethoprim-sulfamethoxazole, and tetracycline (100%), then less sensitive to amikacin and cephalosporine group (71.4%).

### Results of PCR for the detection of certain antibiotic resistance genes

**Figure-1:**
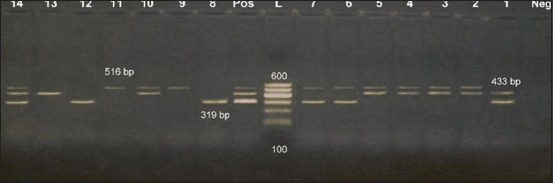
Multiplex polymerase chain reaction detection of antibiotic resistant genes in *Escherichia coli* isolates showing: L: 100 bp DNA ladder. Lanes 1-14: *E. coli* isolates. Lane Pos.: Positive control; amplification of 319 bp represented *aad*B, 433 bp represented *Sul*1, and 516 bp represented *bla*TEM. Lane Neg.: Negative control.

The 14 *E. coli* isolates were screened for presence certain antibiotic resistance determinant genes, the results obtained from Figure-1 revealed that all 14 isolated *E. coli* serotypes which were used in mPCR, carried at least one gene, as 10 carried *bla*TEM (71.4%), 8 *Sul*1 (57.1%), and 6 *aad*B (42.8%), one carried the 3 genes (7.1%) and 8 carried 2 genes (57.1%). These results lead until there were 9 isolates could be considered MDR by an incidence of 64.3%. The previous investigations stated the isolated *E. coli* strains were resistant most frequent to erythromycin (95.83%), cephalothin (62.5%), amikacin (54.17%), kanamycin (45.83%), and gentamicin (41.67%) [25]. While in another one the majority of *E. coli* strains were susceptible to all antimicrobials tested. Resistance to one drug (nalidixic acid, streptomycin, or ampicillin) and resistance to two antimicrobials (ampicillin plus streptomycin and nalidixic acid plus ampicillin) were found in 10 (17.8%) and 2 (3.6%) of the strains, respectively [22].

### Results of PCR for the detection of certain virulence genes

Shiga toxins play a major role in intense inflammatory response and may explain the ability of STEC strains to cause severe illness in affected human and animals. These toxins were expressed by *Stx genes* which constitute two major subfamilies; *Stx*1 and *Stx*2 [26]. Besides that there are other virulent determinants included in *E. coli* pathogenicity; as intimin coded by *eae* gene, which responsible for the attachment and invasion of the organism to mucosa, and *hyl*A gene which expresses the hemolytic activity [27].

**Figure-2:**
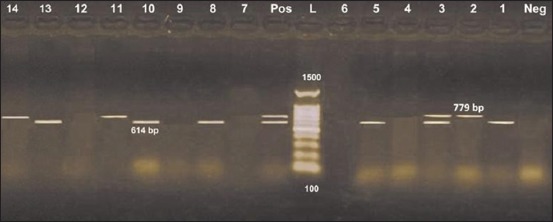
Diplex polymerase chain reaction detection of Shiga toxins genes in *Escherichia coli* isolates showing: L: 100 bp DNA ladder. Lanes 1-14: *E. coli* isolates. Lane Pos.: Positive control; amplification of 614 bp represented *Stx*1, 779 bp represented *Stx*2. Lane Neg.: Negative control.

The results obtained from Figure-2 recognized *Stx*1 gene in 6 isolates (42.8%), two were belonged to both serotypes O125 and O78, while O8 and O158 represented one for each, while *Stx*2 was present in 4 isolates (28.6%), belonged to both O125 and O78. One O125 isolate harbored the two genes, overall, 9 isolates carried either one or the two *Stx* genes (64.3%).

**Figure-3:**
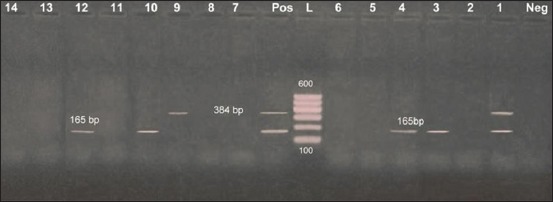
Diplex polymerase chain reaction detection of *hyl*A and *eae* genes in *Escherichia coli* isolates showing: L: 100 bp DNA ladder. Lanes 1-14: *E. coli* isolates. Lane Pos.: Positive control; amplification of 165 bp represented *hyl*A, 384 bp represented *eae*. Lane Neg.: Negative control.

On the other hand, the results obtained from Figure-3 recognized *hyl*A gene in 5 isolates (35.7%), the same of what presented in Figure-2, two were belonged to both serotypes O125 and O78, while one was O8 and O158 represented one for each, while *eae* was present in 2 isolates only (14.3%), belonged to both O8 and O78. One O8 isolate harbored the two genes. These results were near that obtained Oliveira *et al*. [22] who stated that out of 109 STEC isolates, 42 (38.5%) carried *stx*2, 43 (39.5%) carried *stx*1 and *stx*2 sequences, and only 24 (22%) harbored the *stx*1 sequence and no intimin. Furthermore, the prevalence of virulence genes in 49 non-O157 *E. coli* strains, 41 showed detection of *stx*1 (83.1%), 4 *stx*2 (8.2%), 4 both (8.2%), 3 showed detection of *hyl*A (6.1%), and no intimin was detected, Beraldo *et al*. [6], Islam *et al*. [21] characterize *stx*1, *stx*2 in 28.8% and *eae* was found in 14.4% in tested isolates. While in Vu-Khac and Cornick [24] *stx*-positive strains were recovered from 27% of buffaloes and *eae* gene was not detected in buffalo isolates.

Moreover, “*E. coli*” that harboring any gene for Shiga toxins (*stx1* or *stx2*) was detected in 24 (6.61%) of the 363 *E. coli* isolates [25]. On the opposite manner, there were high records as, Borriello *et al*. [23] characterized the 120 *E. coli* isolates for the presence of the virulence factors *Stx*1*, Stx*2, haemolysins, *eae*, and found that *Stx*1 (80%) more frequent than *Stx*2 (27%) while the *eae* and *hyl*A were positive in all isolates.

## Conclusion

The obtained data of our study revealed that the Egyptian buffalo may play role in colonization and dissemination of pathogenic, diarrhegenic *E. coli* and may constitute a prospective human threatening as a source of certain zenotic serotypes infection through milk and meat consumption. The problem is more exacerbating with the marked incidence of multi-antimicrobial resistance isolates, so the attention should be directed to hygienic precautions, as well as careful use of antibiotics in farm animal husbandry.

## Authors’ Contributions

ASH supervised the experiment. STO and ASH shared in the isolation and the antibiotic susceptibility performance, EAF collected the samples and help in antibiotic susceptibility, ASH and EAF identified the isolates by API 20E kit. STO, SMS, and ASH performed PCR, ASH prepared and reviewed the manuscript. All authors read and approved the final manuscript.
